# Global trends and thematic evolution in breast cancer and frailty research: a multi-database bibliometric analysis, 1973–2025

**DOI:** 10.3389/fonc.2026.1975523

**Published:** 2026-10-20

**Authors:** Silu Xiang, Xu Han, Xiaoyi Wang

**Affiliations:** Department of Breast and Thyroid Surgery, Chongqing Key Laboratory of Molecular Oncology and Epigenetics, The First Affiliated Hospital of Chongqing Medical University, Chongqing, China

**Keywords:** bibliometric analysis, breast cancer, frailty, geriatric assessment, geriatric oncology, thematic evolution

## Abstract

**Background:**

Frailty is increasingly recognized as a clinically relevant marker of vulnerability in breast cancer, with implications for treatment tolerance, functional outcomes, and survivorship. This study mapped the global research landscape and thematic evolution of research at the intersection of breast cancer and frailty.

**Methods:**

Publications were retrieved independently from the Web of Science Core Collection (WoSCC), Scopus, and PubMed from database inception through December 31, 2025; publications from 2026 were excluded. After eligibility screening and three-stage cross-database deduplication, 1,003 records were included. Citation analyses were restricted to 591 eligible WoSCC records. Bibliometric analyses were performed using R 4.5.2 with bibliometrix 5.4.0, VOSviewer, and CiteSpace.

**Results:**

Publication output increased over time and reached 115 records in 2025. Segmented Poisson regression estimated annual rate ratios of 1.133 (95% CI, 1.119–1.147) before 2018 and 1.151 (95% CI, 1.105–1.200) after 2018, but the change in slope was not statistically significant (P = 0.465). The United States ranked first in publication output, the Journal of Geriatric Oncology was the most productive journal, and the University of Toronto was the most productive institution. A title/abstract audit of the 1,003-record dataset identified 421 records (41.97%) mentioning both breast cancer and frailty, 83 (8.28%) mentioning breast cancer only, 361 (35.99%) mentioning frailty only, and 138 (13.76%) mentioning neither term category. Keyword and network analyses identified frailty, geriatric assessment, quality of life, comorbidity, treatment, and survivorship as major themes.

**Conclusion:**

Breast cancer and frailty research has expanded into an interdisciplinary field spanning oncology, geriatric medicine, supportive care, rehabilitation, and survivorship. The findings should be interpreted in light of the broader indexing contexts represented in the retrieved set.

## 1 Introduction

Breast cancer remains one of the most common malignancies among women and represents a substantial contributor to the global cancer burden (1). Its clinical management may involve surgery, radiotherapy, chemotherapy, endocrine therapy, and targeted therapy, depending on disease stage and biological characteristics (2). Patients with breast cancer also vary substantially in physiological reserve, functional status, comorbidities, nutritional status, and treatment tolerance. Consequently, conventional oncological factors alone may not fully capture individual vulnerability or treatment-related risk.

Frailty is a clinical syndrome characterized by decreased physiological reserve and increased vulnerability to physiological stressors (3–6). In patients with cancer, frailty is associated with higher risks of treatment-related toxicity, postoperative complications, functional decline, mortality, and poorer quality of life (7–9). In breast cancer specifically, studies have reported substantial variation in frailty prevalence and have increasingly examined its associations with treatment tolerance and clinical outcomes (10). These findings highlight the potential value of frailty as a complementary measure of patient vulnerability in breast cancer care.

Frailty is particularly relevant in older adults, in whom age-related changes, multimorbidity, functional impairment, cognitive problems, and other geriatric vulnerabilities may further complicate cancer treatment. The development of geriatric oncology has therefore promoted the use of frailty assessment and comprehensive geriatric assessment (CGA) in cancer care (11–14). CGA evaluates multiple domains, including functional status, comorbidities, cognition, psychological health, nutrition, medication use, and social support, and can provide information relevant to treatment planning and supportive interventions. As research on breast cancer and frailty has expanded, the field has increasingly involved oncology, geriatric medicine, surgery, nursing, rehabilitation, and supportive care.

Existing reviews have examined the prevalence, assessment, and clinical implications of frailty in patients with cancer and breast cancer (7, 8, 10). However, these reviews have primarily addressed specific clinical questions and do not fully characterize the broader scientific landscape of breast cancer and frailty research. Although bibliometric approaches have been applied to other areas of breast cancer research (15), the literature specifically linking breast cancer and frailty remains insufficiently characterized from a bibliometric perspective. Bibliometric analysis can quantitatively examine publication trends, collaboration networks, intellectual foundations, research hotspots, and thematic evolution (16). Therefore, the present study aimed to characterize the global research landscape, major contributors, collaboration patterns, thematic evolution, and intellectual structure of breast cancer and frailty research from 1973 to 2025.

## 2 Materials and methods

### 2.1 Study design, data sources, and search strategy

This bibliometric mapping study was designed to characterize the research landscape at the intersection of breast cancer and frailty. To strengthen cross-database coverage and reduce dependence on a single indexing source, searches were conducted independently in WoSCC, Scopus, and PubMed and the retrieved records were subsequently harmonized in a unified dataset. The analysis focused on publication patterns, collaboration structures, keyword-based thematic organization, and citation impact rather than on estimating clinical treatment effects.

A systematic literature search was conducted in the Web of Science Core Collection (WoSCC), Scopus, and PubMed databases to identify publications related to breast cancer and frailty. These databases were selected because they provide complementary coverage of biomedical, multidisciplinary, and citation-indexed publications. Searches were conducted on May 25, 2026, across all three databases, and covered publications indexed from database inception through December 31, 2025. Records published in 2026 were excluded during data cleaning, as publications from that year were still incompletely indexed at the time of retrieval and could introduce time-truncation bias into the analysis of annual trends.

The search strategy was designed around two core concepts: breast cancer and frailty. To improve topic specificity, terms related to statistical frailty models, such as “frailty model”, “shared frailty” and “unobserved heterogeneity”, were excluded. The PubMed search strategy was as follows:

(“Breast Neoplasms”[Mesh] OR “breast cancer”[Title/Abstract] OR “breast neoplasm”[Title/Abstract] OR “breast neoplasms”[Title/Abstract] OR “breast carcinoma”[Title/Abstract] OR “breast carcinomas”[Title/Abstract] OR “mammary cancer”[Title/Abstract] OR “mammary carcinoma”[Title/Abstract] OR “mammary neoplasm”[Title/Abstract] OR “mammary neoplasms”[Title/Abstract]) AND (“Frailty”[Mesh] OR frail*[Title/Abstract] OR frailty[Title/Abstract] OR “frailty syndrome”[Title/Abstract]) NOT (“frailty model”[Title/Abstract] OR “frailty models”[Title/Abstract] OR “shared frailty”[Title/Abstract] OR “random frailty”[Title/Abstract] OR “unobserved heterogeneity”[Title/Abstract]).

The WoSCC search strategy was:

TS = ((“breast cancer” OR “breast neoplasm*” OR “breast carcinoma*” OR “mammary cancer” OR “mammary carcinoma*” OR “mammary neoplasm*”) AND (frail* OR frailty OR “frailty syndrome”)) NOT TS = (“frailty model” OR “frailty models” OR “shared frailty” OR “random frailty” OR “unobserved heterogeneity”).

The Scopus search strategy was:

TITLE-ABS-KEY((“breast cancer” OR “breast neoplasm*” OR “breast carcinoma*” OR “mammary cancer” OR “mammary carcinoma*” OR “mammary neoplasm*”) AND (frail* OR frailty OR “frailty syndrome”)) AND NOT TITLE-ABS-KEY(“frailty model” OR “frailty models” OR “shared frailty” OR “random frailty” OR “unobserved heterogeneity”).

Only English-language publications were considered. Articles and reviews were retained for bibliometric analysis, while non-research document types were removed during screening. The initial retrieval yielded 1,913 records, including 630 from WoSCC, 812 from Scopus, and 471 from PubMed. Record identification, screening, eligibility assessment, and inclusion were documented using a PRISMA 2020-informed flow diagram adapted to the bibliometric design (17).

To evaluate topical specificity, title and abstract fields were audited using prespecified breast-cancer expressions and frail* variants. Each record was assigned to one of four mutually exclusive categories: both breast cancer and frailty, breast cancer only, frailty only, or neither term category in the title and/or abstract. This audit was performed for both the complete 1,003-record dataset and the 591-record WoSCC citation subset. Separately, the titles and abstracts of the 10 most globally cited WoSCC records were assessed manually using an explicit three-level framework: directly central when the breast cancer-frailty relationship was the primary research question; contextual when a record directly addressed cancer-related frailty, geriatric oncology, or breast cancer-related vulnerability without examining the joint relationship; and peripheral when the breast cancer-frailty interface was absent from the title and abstract and eligibility arose through indexing fields or broader contextual mentions. These audits characterized thematic specificity but did not override eligibility under the predefined database-level retrieval strategy.

### 2.2 Record export and dataset integration

Bibliographic records were exported separately from each database, with WoSCC records containing full records and cited references, Scopus records exported in structured format, and PubMed records exported in Citation Manager format. All records were subsequently imported into R software (version 4.5.2) and transformed into a unified bibliographic data structure using the bibliometrix package (version 5.4.0) (18).

At this stage, records from the three databases were first merged without relying on automatic cross-database deduplication. This allowed year filtering, document type screening, and duplicate removal to be carried out in a transparent and reproducible manner. Field names, publication years, document types, titles, DOIs, author information, institutional affiliations, countries or regions, journals, and keywords were checked and standardized where feasible before subsequent analyses.

Database provenance flags were retained during integration and deduplication. The final records were assigned to seven mutually exclusive source combinations (WoSCC only, Scopus only, PubMed only, WoSCC plus Scopus, WoSCC plus PubMed, Scopus plus PubMed, or all three databases). These combinations were used to quantify database overlap and unique added coverage and to construct **Supplementary Figure S2**.

### 2.3 Year filtering, document type selection, and deduplication

After records from the three databases were merged, publications published after 2025 or lacking valid publication-year information were excluded. Document-type screening was then performed, with articles and reviews retained and non-target publication types excluded.

Duplicate removal was performed in three sequential stages across the complete integrated library. First, DOI strings were converted to a consistent case and DOI URL/prefixes and spaces were removed; exact normalized DOI matches were then collapsed. Second, titles were converted to a consistent case, punctuation was removed, whitespace was collapsed, and exact normalized title-plus-publication-year matches were collapsed irrespective of DOI availability. Third, remaining potential title matches were generated with Jaro–Winkler similarity (stringdist::stringsim, method = “jw”) (19). Pairs from the same publication year with similarity ≥0.90 were reviewed, and same-first-author pairs from adjacent years were additionally screened at similarity ≥0.95. Every fuzzy candidate was manually classified as DUPLICATE or KEEP; no record was deleted automatically on the basis of fuzzy similarity. When duplicate versions were consolidated, the representative record was selected in the order WoSCC, Scopus, and PubMed, favoring the most complete metadata and filling selected blank fields from duplicate versions. The full thresholds, rules, and stage-specific counts are supplied in **Supplementary Data Sheet 1**.

### 2.4 Bibliometric analyses

Bibliometric analyses were performed on the final integrated dataset of 1,003 publications to describe the research landscape of breast cancer and frailty. The analyses included annual scientific production, country or regional distribution, international collaboration patterns, productive authors, active journals, contributing institutions, keyword frequency, keyword co-occurrence, citation impact, and thematic evolution.

Annual publication output was used to characterize the temporal development of the field. Country or regional productivity was assessed by publication counts. Single-country publications (SCP) and multiple-country publications (MCP) were calculated to describe domestic and international collaboration patterns, and the MCP ratio was used as an indicator of international collaboration intensity. Productive authors, journals, and institutions were ranked by the number of publications to identify major contributors and core publication venues in this field.

To evaluate the proposed change in publication growth after 2018, annual counts were modeled using segmented Poisson regression with terms for time, an indicator after 2018, and years elapsed after 2018. The Pearson dispersion ratio was examined *a priori*, with negative binomial regression planned if the ratio exceeded 1.5. Annual incidence rate ratios (IRRs), 95% confidence intervals, and the P value for the post-2018 slope change were reported.

For the top 10 countries, MCP proportions were accompanied by Wilson 95% confidence intervals. Country comparisons were treated as descriptive because internationally coauthored papers contribute to more than one country total. The 591-record WoSCC citation subset was compared with the full 1,003-record dataset by publication year, harmonized country, and journal; Spearman rank correlations were calculated for the corresponding distributions, and median publication year and recent-period proportions were also compared.

### 2.5 Keyword preprocessing and hotspot analysis

Keyword analysis was conducted to identify major research topics and their distribution in the field of breast cancer and frailty. Keywords were extracted from author keywords, index keywords, and available subject-heading fields. Normalization converted terms to lowercase, replaced underscores and hyphens with spaces, removed punctuation, and collapsed repeated whitespace. A prespecified conservative dictionary then merged only established lexical variants and abbreviations, including breast-cancer variants, frail/frailty variants, geriatric-assessment variants, older-adult variants, quality-of-life variants, and survival abbreviations. General methodological or demographic indexing terms with limited thematic value were removed using a documented stopword list. Residual variants were retained whenever equivalence could not be established with confidence. The complete dictionary, observed raw-to-clean mapping, and the number of variants merged into every harmonized target are provided in **Supplementary Data Sheet 1**.

Based on the cleaned keyword set, keyword frequency tables, a word cloud, a treemap, and a ranked bar plot were generated. The word cloud provided an intuitive overview of high-frequency keywords, the treemap represented their relative contribution by area, and the bar plot displayed the exact frequencies of the top 20 terms. These visualizations show thematic prominence rather than semantic relationships among terms.

### 2.6 Collaboration and co-occurrence network analysis

VOSviewer was used to construct and visualize collaboration and co-occurrence networks (20). For country or regional collaboration analysis, the analysis type was set to co-authorship, and the unit of analysis was set to countries or regions. Full counting was used. In the resulting network, node size represented publication output, links indicated collaborative relationships, link thickness reflected collaboration strength, and colors represented collaboration clusters.

Keyword co-occurrence analysis was performed using the cleaned keyword data. A document–keyword co-occurrence matrix was constructed and imported into VOSviewer. Full counting and association-strength normalization were used. The final display applied a minimum occurrence threshold of 20, which was met by 191 cleaned keywords. In the keyword network, node size represented occurrence frequency, links represented co-occurrence relationships, and colors indicated VOSviewer clusters. Because VOSviewer does not output a CiteSpace-style silhouette statistic, a separate R/Louvain robustness analysis (21) was performed on the full cleaned graph after retaining edges with co-occurrence weight ≥2; its modularity was Q = 0.2560, indicating substantial thematic overlap. Accordingly, VOSviewer clusters were interpreted as exploratory thematic groupings rather than sharply separated categories.

### 2.7 CiteSpace keyword clustering and burst analysis

CiteSpace was used to further explore thematic clustering and the temporal evolution of research hotspots (22). Two time windows were defined: 1995–2025 for the overall cluster and burst analysis and 2016–2025 for the recent-period analysis. The overall window began in 1995 because the literature was too sparse before that year to support a stable annual-slice keyword network, whereas the full annual production series remained 1973–2025. The time slice was set to 1 year.

Term sources included titles, abstracts, author keywords, and index keywords. Network link strength was calculated using cosine similarity with within-slice links. Node selection used the g-index with k = 25; the link retaining factor was 2.5, the maximum links per node was 10, look-back years was 5, and e = 1.0. No pruning option was applied. Keyword clustering was performed with log-likelihood ratio (LLR) labels, and burst detection identified terms showing sharp increases in attention during specific periods. LLR labels were treated as algorithm-generated descriptors and interpreted together with the terms and publications within each cluster.

### 2.8 Citation analysis

Citation analyses were restricted to 591 eligible records from WoSCC because citation coverage, cited-reference formats, and counting rules differ across databases. Of the 630 records initially retrieved from WoSCC, 38 publications from 2026 and one record excluded during document-type screening were removed in accordance with the eligibility criteria applied to the main analysis. PubMed records were not used for citation relationship analysis because standardized cited-reference metadata are not available in the same form as in WoSCC. Global citation counts were retrieved from WoSCC on May 25, 2026.

Two citation indicators were considered. Global citations referred to the total number of citations received by a publication in WoSCC and were used to describe its broader citation impact. Local citations referred to the number of times a publication was cited by other records within the 591-record eligible WoSCC citation-analysis subset and were used to describe its field-specific influence within the retrieved literature. The top globally and locally cited publications were ranked separately. AI-assisted code suggestions were reviewed and independently executed by the authors, and all analytical outputs were verified against the underlying bibliographic data.

## 3 Results

### 3.1 Literature retrieval results

A total of 1,913 records were initially retrieved from the three databases, including 630 records from WoSCC, 812 from Scopus, and 471 from PubMed. After removal of 125 records published after 2025 or lacking valid publication-year information, 1,788 records remained. Document type screening excluded 13 non-target publications, leaving 1,775 records for deduplication.

Deduplication removed 610 exact DOI duplicates, 153 additional duplicates identified by exact normalized title and publication year across the full library, and 9 manually confirmed fuzzy-title duplicates. The final integrated final integrated dataset therefore comprised 1,003 unique publications. The complete selection process is shown in **Figure 1**.

**Figure 1:**
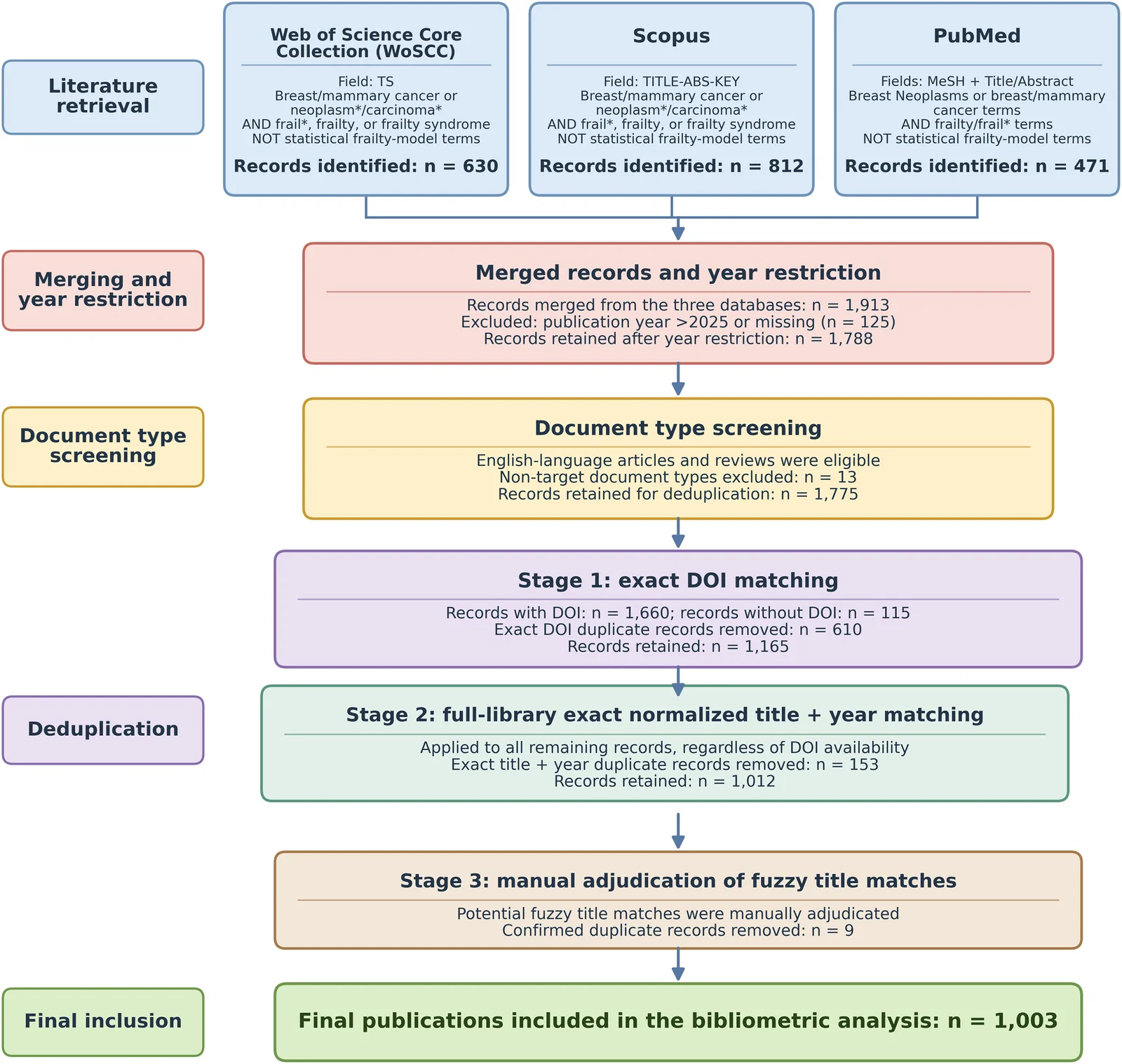
Literature identification, screening, deduplication, and inclusion process. English-language records were retrieved independently from WoSCC, Scopus, and PubMed using database-specific search strategies for breast cancer and frailty, with statistical frailty-model terminology excluded. After year and document-type screening, deduplication removed 610 exact DOI duplicates, 153 exact normalized title-and-year duplicates, and 9 manually confirmed fuzzy-title duplicates, yielding 1,003 unique publications.

Database-overlap analysis showed 321 records present in all three databases, 324 in Scopus only, 205 in WoSCC only, 26 in PubMed only, 62 in Scopus plus PubMed, 52 in WoSCC plus Scopus, and 13 in WoSCC plus PubMed. Overall, 759 final records occurred in Scopus, 591 in WoSCC, and 422 in PubMed; 412 records (41.1%) were not present in WoSCC. These results are shown in **Supplementary Figure S2** and **Supplementary Data Sheet 1**.

The title/abstract audit of the final 1,003 records classified 421 (41.97%) as mentioning both breast cancer and frailty, 83 (8.28%) as breast cancer only, 361 (35.99%) as frailty only, and 138 (13.76%) as neither term category. Thus, 504 records (50.25%) explicitly mentioned breast cancer and 782 (77.97%) explicitly mentioned frailty in the title and/or abstract. Within the 591-record WoSCC subset, 338 (57.19%) mentioned both, 40 (6.77%) breast cancer only, 169 (28.60%) frailty only, and 44 (7.45%) neither; the corresponding breast-cancer and frailty totals were 378 (63.96%) and 507 (85.79%), respectively. Records not explicitly mentioning both concepts could have been retrieved through database indexing fields or broader contextual mentions.

### 3.2 Annual publication trends

The annual scientific production of studies on breast cancer and frailty from 1973 to 2025 is shown in **Figure 2**. Publication output remained limited before 2000, with most years contributing fewer than five publications. Output then increased gradually, reaching 8 publications in 2000, 11 in 2004, 17 in 2006, 20 in 2010, and 31 in 2014.

**Figure 2:**
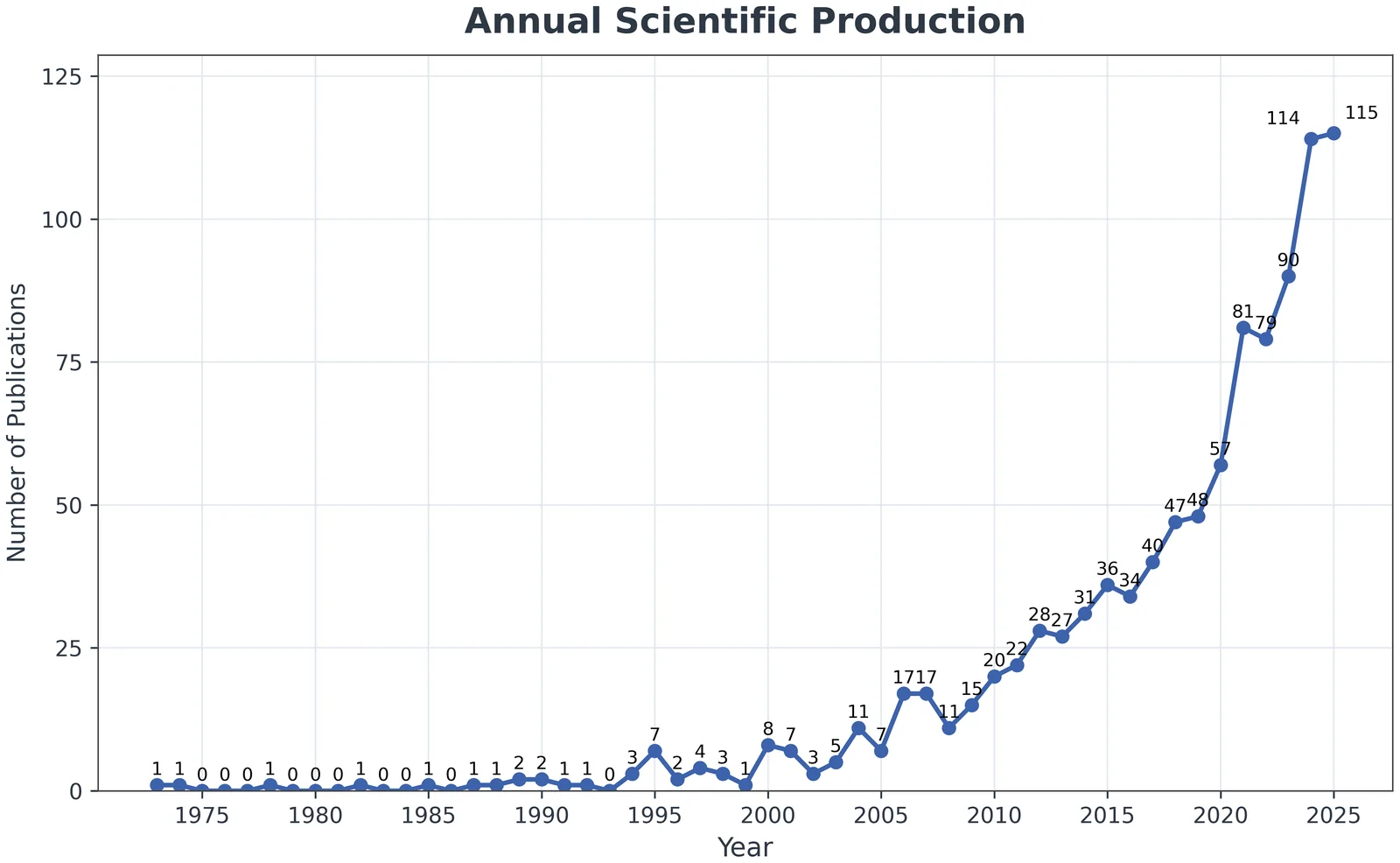
Annual scientific production of publications related to breast cancer and frailty from 1973 to 2025. Publication counts increased over time and reached 115 in 2025. Segmented Poisson regression estimated annual rate ratios of 1.133 before 2018 and 1.151 after 2018, but the slope change was not statistically significant (P = 0.465).

Annual counts were visually higher in the later years, increasing from 47 publications in 2018 to 57 in 2020, 81 in 2021, 90 in 2023, 114 in 2024, and 115 in 2025. In segmented Poisson regression, the annual rate ratio was 1.133 (95% CI, 1.119–1.147) before 2018 and 1.151 (95% CI, 1.105–1.200) after 2018. The slope change after 2018 was not statistically significant (P = 0.465); therefore, the results support a sustained increase over time but not a discrete acceleration beginning in 2018.

### 3.3 Country distribution and collaboration patterns

**Table 1** summarizes the top 10 countries or regions ranked by publication output and international collaboration. The United States contributed the largest number of publications (n = 355), followed by Italy (n = 144), the United Kingdom (n = 140), France (n = 82), the Netherlands (n = 66), Canada (n = 61), Belgium (n = 57), China (n = 57), Germany (n = 54), and Spain (n = 41). The harmonized country labels show that research activity was concentrated principally in North America and Europe, with China also represented among the ten leading contributors.

**Table 1:** Top 10 countries/regions ranked by publication output and international collaboration in breast cancer and frailty research.

Rank	Country/Region	Publications	SCP	MCP	MCP (%)	MCP 95% CI (%)
1	United States	355	268	87	24.5	20.3–29.2
2	Italy	144	76	68	47.2	39.2–55.3
3	United Kingdom	140	77	63	45.0	37.0–53.3
4	France	82	46	36	43.9	33.7–54.7
5	Netherlands	66	35	31	47.0	35.4–58.8
6	Canada	61	29	32	52.5	40.2–64.5
7	Belgium	57	19	38	66.7	53.7–77.5
8	China	57	36	21	36.8	25.5–49.8
9	Germany	54	17	37	68.5	55.3–79.3
10	Spain	41	18	23	56.1	41.0–70.1

SCP, single-country publications; MCP, multiple-country publications. MCP percentages are accompanied by Wilson 95% confidence intervals and are interpreted descriptively. Country names were harmonized before analysis; England, Scotland, Wales, Northern Ireland, UK, U.K., and Great Britain were consolidated under United Kingdom, and duplicate country occurrences within a publication were collapsed.

The top 20 contributing countries and regions after geographic harmonization are shown in **Supplementary Figure S1**.

Patterns of international collaboration were summarized descriptively using MCP proportions with Wilson 95% confidence intervals. The point estimate was 24.5% (95% CI, 20.3%–29.2%) for the United States. The corresponding estimates were 47.2% (39.2%–55.3%) for Italy, 45.0% (37.0%–53.3%) for the United Kingdom, 43.9% (33.7%–54.7%) for France, 47.0% (35.4%–58.8%) for the Netherlands, 52.5% (40.2%–64.5%) for Canada, 66.7% (53.7%–77.5%) for Belgium, 36.8% (25.5%–49.8%) for China, 68.5% (55.3%–79.3%) for Germany, and 56.1% (41.0%–70.1%) for Spain. These values are descriptive estimates; no pairwise claims of statistically significant country differences were made.

### 3.4 Top authors, journals, and institutions in breast cancer and frailty research

**Figure 3** presents the most productive authors, journals, and institutions. Wildiers H ranked first with 24 publications, followed by Cheung KL (n = 20), Cohen HJ (n = 19), Hurria A and Wyld L (n = 17 each), Muss HB (n = 16), Sousa P (n = 15), and Cunha M, Extermann M, and Horgan K (n = 14 each). These contributors represent sustained activity across geriatric oncology, treatment decision-making, supportive care, and frailty-related outcomes.

**Figure 3:**
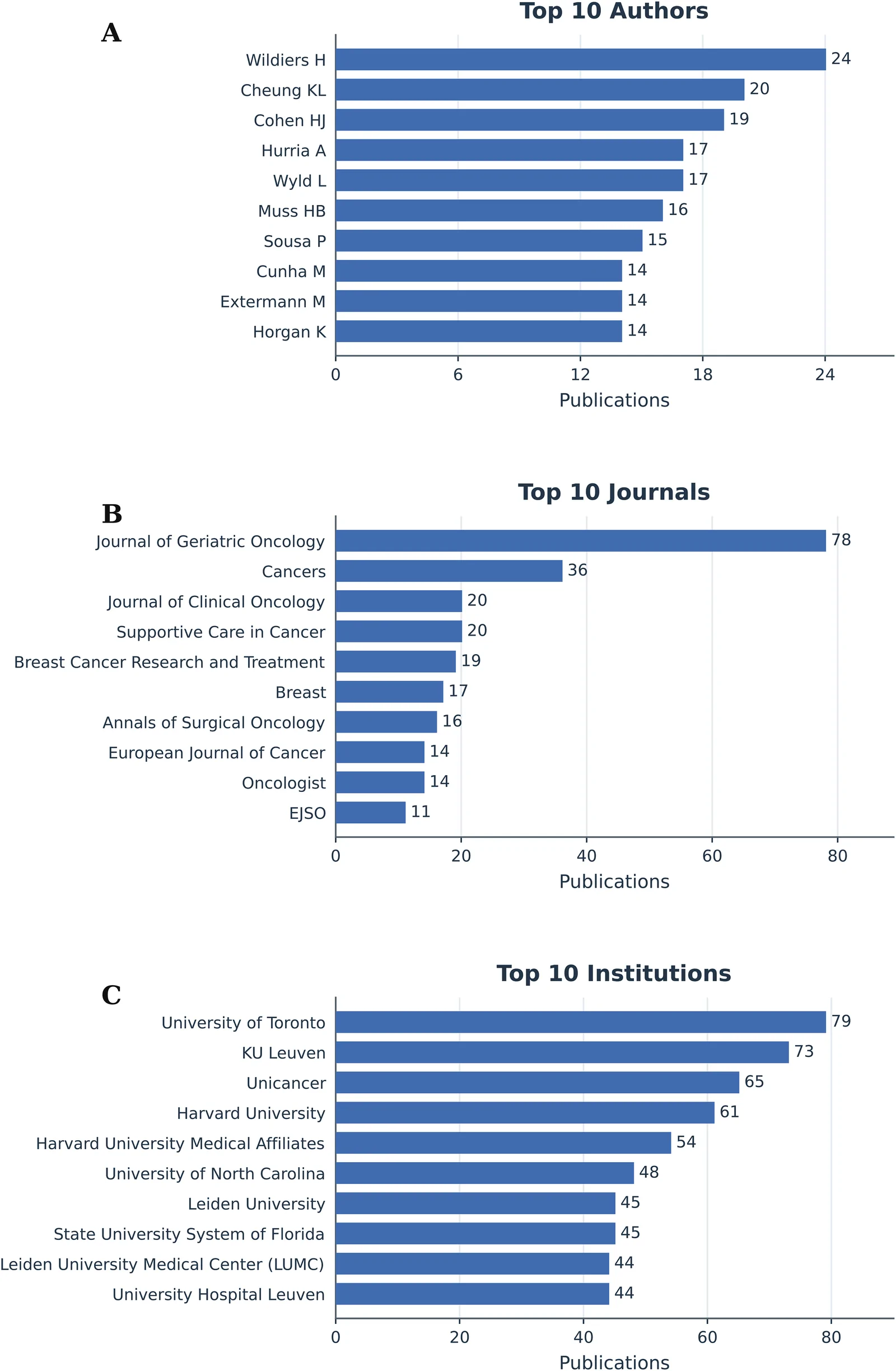
Productive contributors and publication sources in breast cancer and frailty research. **(A)** Top 10 authors ranked by number of publications. **(B)** Top 10 journals ranked by publication output. **(C)** Top 10 institutions ranked by number of publications. Bar length represents the number of publications.

The Journal of Geriatric Oncology was the most productive journal, with 78 publications, followed by Cancers (n = 36), Journal of Clinical Oncology and Supportive Care in Cancer (n = 20 each), Breast Cancer Research and Treatment (n = 19), Breast (n = 17), Annals of Surgical Oncology (n = 16), European Journal of Cancer and The Oncologist (n = 14 each), and European Journal of Surgical Oncology (n = 11). This distribution reflects the interdisciplinary nature of the field.

The University of Toronto ranked first in institutional productivity with 79 publications, followed by KU Leuven (n = 73), UNICANCER (n = 65), Harvard University (n = 61), Harvard Medical Affiliates (n = 54), the University of North Carolina (n = 48), Leiden University and the State University System of Florida (n = 45 each), and Leiden University Medical Center and University Hospitals Leuven (n = 44 each). The institutional distribution reflects the contribution of major academic and clinical centers in oncology and geriatric medicine.

### 3.5 Keyword distribution and research hotspots

To identify major research topics, cleaned keywords were analyzed by frequency and visualized using a word cloud, a treemap, and a top-20 bar plot (**Figures 4A–C**). The most prominent terms concerned breast cancer, frailty, geriatric assessment, quality of life, comorbidity, aging, chemotherapy, and outcomes, indicating sustained attention to vulnerability assessment, treatment decision-making, and patient-centered outcomes.

**Figure 4:**
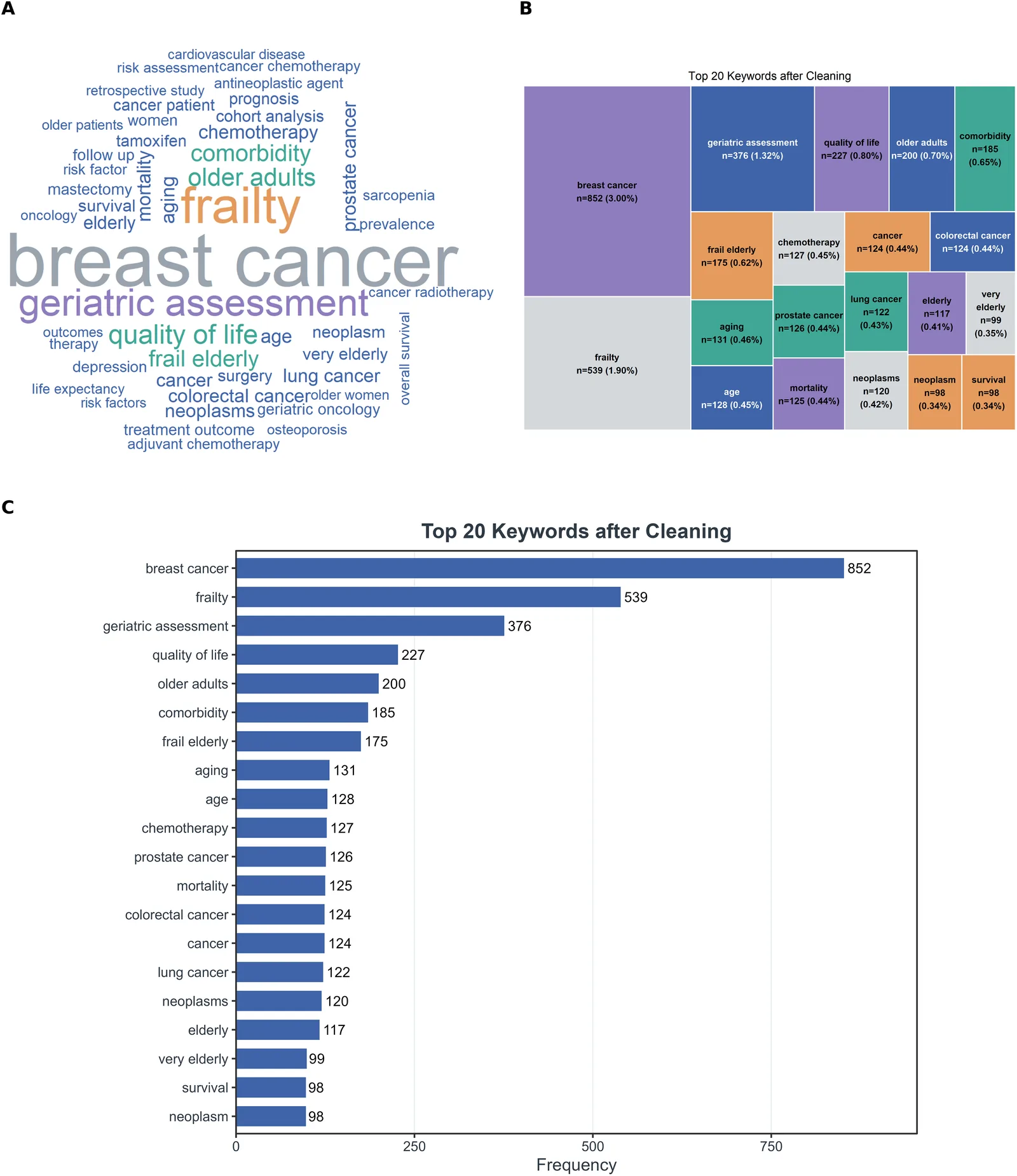
Distribution of the top cleaned keywords in the 1,003-record final harmonized dataset. **(A)** Word cloud, where font size represents frequency. **(B)** Treemap of the top 20 terms, where rectangle size represents relative frequency. **(C)** Ranked bar plot showing exact frequencies for the top 20 terms. Search concepts were expected to rank highly; residual variants such as “neoplasm” and “neoplasms” were retained as separately indexed forms.

Across the three complementary displays, “breast cancer” and “frailty” were the two most frequent terms, followed by “geriatric assessment”. Other prominent terms included “quality of life”, “older adults”, “comorbidity”, “frail elderly”, “aging”, “age”, and “chemotherapy”. The dominance of the two search concepts was expected and was interpreted together with the surrounding clinical and geriatric topics.

The ranked frequencies showed 852 occurrences of “breast cancer”, 539 of “frailty”, 376 of “geriatric assessment”, 227 of “quality of life”, 200 of “older adults”, 185 of “comorbidity”, 175 of “frail elderly”, 131 of “aging”, 128 of “age”, and 127 of “chemotherapy”. Other frequent terms included prostate cancer (n = 126), mortality (n = 125), colorectal cancer and cancer (n = 124 each), lung cancer (n = 122), neoplasms (n = 120), elderly (n = 117), very elderly (n = 99), and survival and neoplasm (n = 98 each). The presence of generic neoplasm terms and non-breast cancer terms reflects broader geriatric-oncology and indexing contexts within the retrieved set rather than disease-specific hotspots alone.

Taken together, the keyword analysis identified three broad domains: frailty identification and geriatric assessment; treatment, toxicity, mortality, and survival; and health status and patient-centered outcomes, including quality of life and comorbidity. Residual variants such as “neoplasm” and “neoplasms” were retained as separately indexed forms and are acknowledged as a remaining preprocessing limitation.

### 3.6 Country collaboration and keyword co-occurrence networks based on VOSviewer

To further characterize international collaboration patterns and the conceptual structure of breast cancer and frailty research, country collaboration and keyword co-occurrence networks were visualized using VOSviewer (**Figure 5**). In the country collaboration network shown in **Figure 5A**, node size represents publication output, links indicate collaborative relationships between countries or regions, link thickness reflects collaboration strength, and colors represent network clusters. **Figure 5B** presents the keyword co-occurrence network, in which node size represents keyword frequency, links indicate co-occurrence relationships, and colors represent thematic clusters.

**Figure 5:**
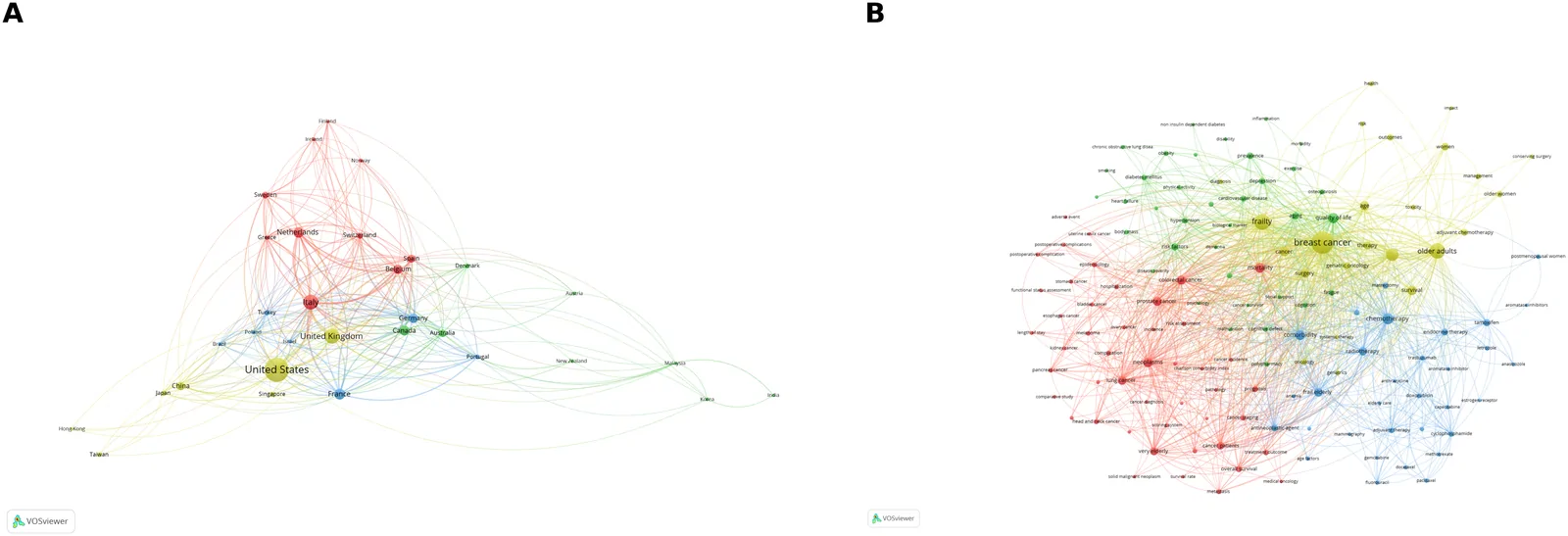
VOSviewer collaboration and keyword co-occurrence networks. **(A)** Country collaboration network based on harmonized geographic labels; node size represents publication output, links indicate collaboration, link thickness reflects collaboration strength, and colors indicate clusters. **(B)** Keyword co-occurrence network generated with full counting and a minimum occurrence threshold of 20; 191 cleaned keywords met the threshold. Node size represents occurrence frequency, links indicate co-occurrence, and colors indicate exploratory thematic clusters.

The country collaboration network showed visible international connectivity, particularly among research groups in Europe and North America. The United States, Italy, the United Kingdom, France, Germany, Canada, the Netherlands, Spain, and Australia were among the more visible contributors. These patterns are descriptive and should not be interpreted as statistically significant differences in collaboration propensity.

The keyword co-occurrence network further revealed the conceptual structure and major thematic relationships within the field. Keywords such as “breast cancer”, “frailty”, “aged”, “chemotherapy”, “quality of life”, “comorbidity”, “mortality”, “survival”, “geriatric assessment”, and “elderly patients” were represented by relatively large nodes and showed multiple visible co-occurrence links. Larger nodes indicate higher keyword frequencies, while dense links among nodes suggest that these topics frequently appeared together in the literature.

At the minimum occurrence threshold of 20, 191 cleaned keywords were displayed. The co-occurrence pattern suggested broad treatment-related, geriatric-assessment, and patient-outcome domains. However, the separate R/Louvain robustness analysis yielded modularity Q = 0.2560, indicating substantial cross-cluster overlap. We therefore interpret the colored VOSviewer groups as exploratory thematic groupings rather than discrete or independently validated categories.

Overall, the country network showed visible cross-national collaboration, particularly among European and North American research groups. The keyword network identified treatment-related topics, geriatric assessment and frailty, and clinical or patient-centered outcomes as the major thematic groupings in the analyzed literature.

### 3.7 CiteSpace keyword clustering and burst analysis

CiteSpace clustering and burst detection were conducted for a recent window (2016–2025; **Figure 6**) and an overall window (1995–2025; **Figure 7**). The recent network had modularity Q = 0.4069 and weighted mean silhouette S = 0.7112; the overall network had Q = 0.4379 and S = 0.7639. These values indicate moderate modular separation and generally acceptable within-cluster consistency, while also showing that thematic overlap remained.

**Figure 6:**
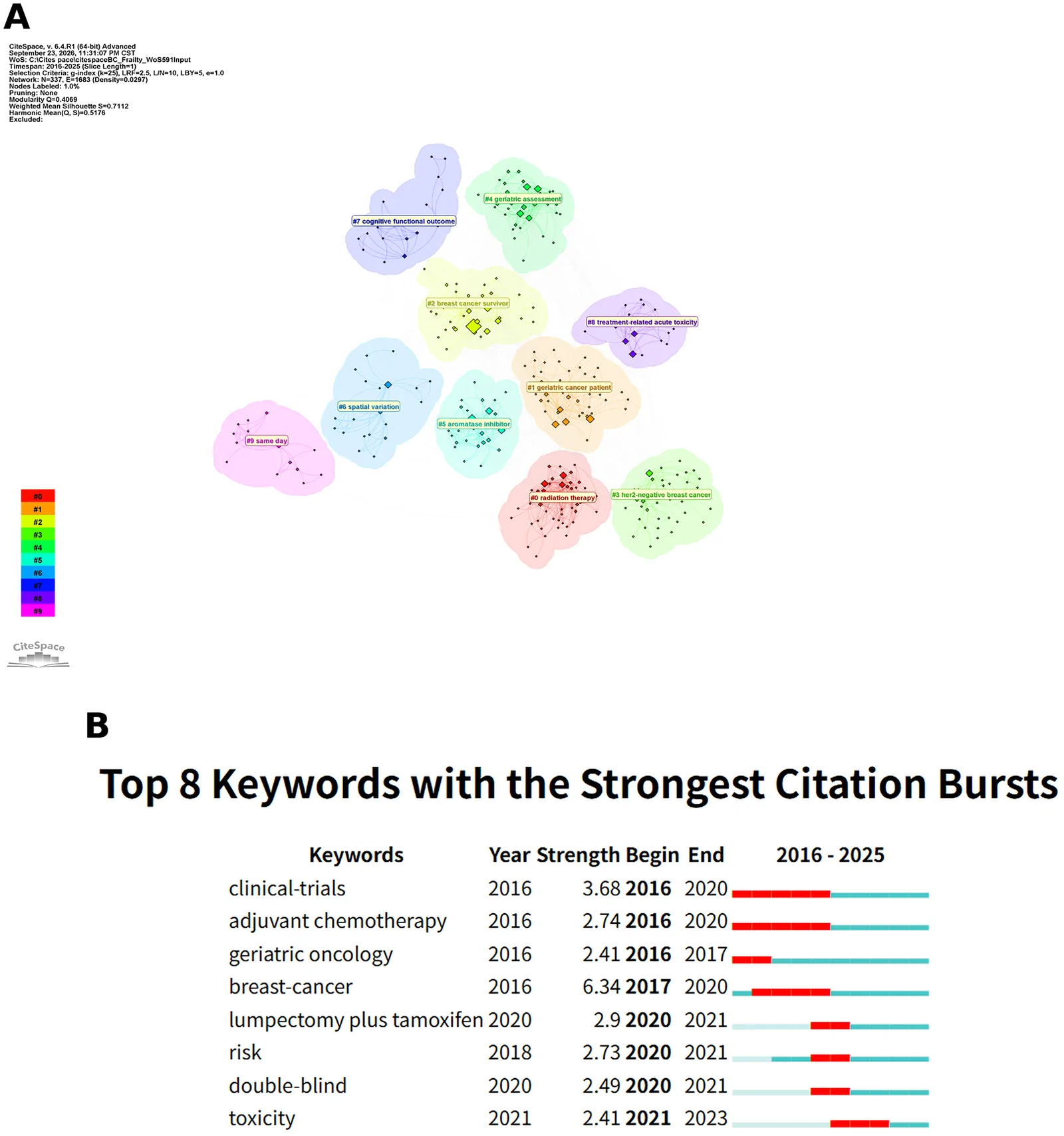
Recent thematic clusters and keyword citation bursts identified by CiteSpace for 2016–2025. **(A)** Keyword cluster map with LLR-generated labels (modularity Q = 0.4069; weighted mean silhouette S = 0.7112). **(B)** Eight keywords with the strongest citation bursts; red segments indicate burst periods. LLR labels are algorithm-generated descriptors and should be interpreted with the terms and publications within each cluster.

**Figure 7:**
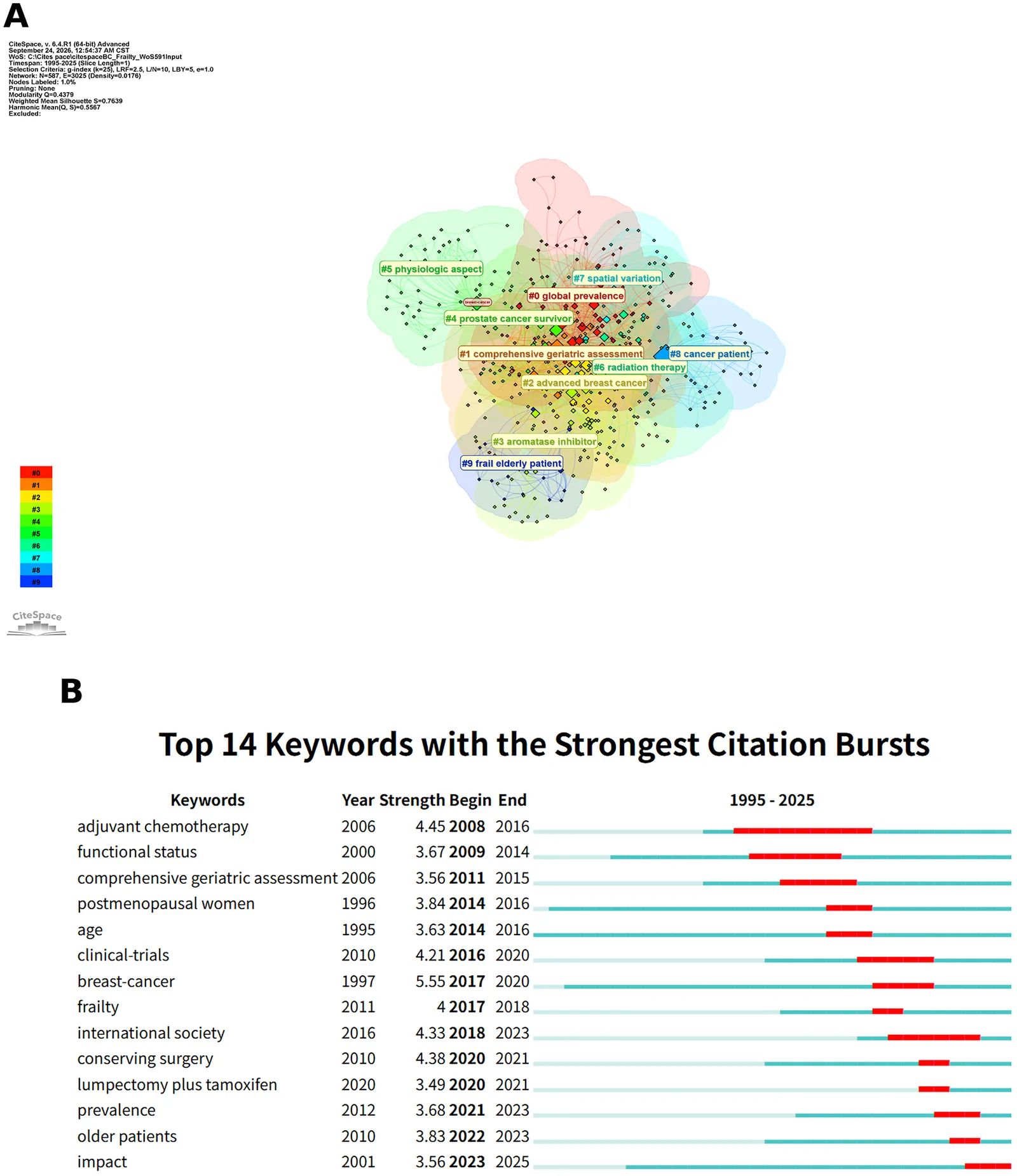
Overall thematic clusters and keyword citation bursts identified by CiteSpace for 1995–2025. **(A)** Keyword cluster map with LLR-generated labels (modularity Q = 0.4379; weighted mean silhouette S = 0.7639). **(B)** Fourteen keywords with the strongest citation bursts; red segments indicate burst periods. The overall CiteSpace window begins in 1995 because earlier annual output was too sparse for a stable sliced keyword network; the descriptive annual series in **Figure 2** remains 1973–2025.

The 2016–2025 LLR cluster map contained 11 displayed clusters. The largest labels included #0 radiation therapy, #1 geriatric cancer patient, #2 breast cancer survivor, #3 HER2-negative breast cancer, #4 geriatric assessment, #5 aromatase inhibitor, #6 spatial variation, #7 cognitive functional outcome, #8 treatment-related acute toxicity, #9 same day, and #10 metformin. Collectively, these clusters point to treatment selection, survivorship, geriatric assessment, cognitive and toxicity outcomes, and variation in care. Because LLR labels are selected statistically from cluster terms, they should be read as concise labels rather than as exhaustive descriptions of each cluster.

The recent-period burst analysis identified eight terms. “Breast-cancer” showed the strongest burst (strength = 6.34; 2017–2020). “Clinical-trials” (3.68; 2016–2020), “adjuvant chemotherapy” (2.74; 2016–2020), and “geriatric oncology” (2.41; 2016–2017) characterized the earlier part of the window. “Lumpectomy plus tamoxifen” (2.90; 2020–2021), “risk” (2.73; 2020–2021), “double-blind” (2.49; 2020–2021), and “toxicity” (2.41; 2021–2023) marked later short-duration bursts, suggesting growing attention to treatment burden, study design, and tolerability.

The 1995–2025 LLR cluster map showed a broader long-term thematic structure. Major displayed labels included #0 global prevalence, #1 comprehensive geriatric assessment, #2 advanced breast cancer, #3 aromatase inhibitor, #4 prostate cancer survivor, #5 physiologic aspect, #6 radiation therapy, #7 spatial variation, #8 cancer patient, #9 frail elderly patient, #10 cognitive functional outcome, #11 competitive reverse transcription-polymerase chain reaction, and #12 age factor. The mixture of breast cancer-specific and broader oncology labels is consistent with the retrieval audit and should be interpreted from the underlying cluster records rather than from labels alone.

Fourteen terms were displayed in the 1995–2025 burst analysis. Earlier bursts included “adjuvant chemotherapy” (strength = 4.45; 2008–2016), “functional status” (3.67; 2009–2014), and “comprehensive geriatric assessment” (3.56; 2011–2015). Later bursts included “clinical-trials” (4.21; 2016–2020), “breast-cancer” (5.55; 2017–2020), “frailty” (4.00; 2017–2018), “international society” (4.33; 2018–2023), “conserving surgery” (4.38; 2020–2021), “older patients” (3.83; 2022–2023), and “impact” (3.56; 2023–2025). The nonspecific label “international society” was interpreted cautiously.

Together, the two CiteSpace analyses suggest a shift from treatment-centered themes toward geriatric assessment, frailty, tolerability, functional and cognitive outcomes, and survivorship. This interpretation is based on the joint pattern of clusters, burst timing, and underlying terms rather than on any single LLR label.

### 3.8 Citation analysis

Global and local citation analyses were restricted to the 591 eligible WoSCC records, representing 58.9% of the 1,003-record integrated dataset, and are presented in **Tables 2** and **3**. The full dataset and WoSCC subset had the same median publication year (2020); 62.9% and 64.3%, respectively, were published in 2018–2025. Spearman correlations between the full and subset distributions were 0.986 by publication year, 0.911 by country, and 0.592 by journal. Thus, the subset closely reproduced the temporal and country patterns but showed only moderate journal-level alignment. Citation findings are therefore reported explicitly as WoSCC-specific rather than as fully representative of all three databases.

**Table 2:** Top 10 globally cited records captured by the predefined search strategy within the 591-record WoSCC subset.

No.	First author	Yr	Article title	Journal	Global cites	Targeted manual assessment
1	Handforth C	2015	The prevalence and outcomes of frailty in older cancer patients: a systematic review	Annals of Oncology	838	Contextual
2	Tan BL	2018	Antioxidant and oxidative stress: a mutual interplay in age-related diseases	Frontiers in Pharmacology	812	Peripheral
3	Lamberts SWJ	1997	The endocrinology of aging	Science	728	Peripheral
4	Ethun CG	2017	Frailty and cancer: implications for oncology surgery, medical oncology, and radiation oncology	CA: A Cancer Journal for Clinicians	500	Contextual
5	Sedrak MS	2021	Older adult participation in cancer clinical trials: a systematic review of barriers and interventions	CA: A Cancer Journal for Clinicians	396	Contextual
6	Chen XJ	2020	Consumption of ultra-processed foods and health outcomes: a systematic review of epidemiological studies	Nutrition Journal	388	Peripheral
7	Schuurmans H	2004	Old or frail: what tells us more?	The Journals of Gerontology Series A: Biological Sciences and Medical Sciences	380	Peripheral
8	Hurria A	2014	Designing therapeutic clinical trials for older and frail adults with cancer: U13 conference recommendations	Journal of Clinical Oncology	276	Contextual
9	Kiecolt-Glaser JK	2014	Yoga’s impact on inflammation, mood, and fatigue in breast cancer survivors: a randomized controlled trial	Journal of Clinical Oncology	265	Contextual
10	Wildiers H	2013	End points and trial design in geriatric oncology research: a joint European Organisation for Research and Treatment of Cancer–Alliance for Clinical Trials in Oncology–International Society of Geriatric Oncology position article	Journal of Clinical Oncology	234	Contextual

Global citation counts indicate broad WoSCC impact and do not necessarily establish thematic specificity to the breast cancer-frailty interface. Targeted manual title/abstract assessment classified records as directly central, contextual, or peripheral; six were contextual, four were peripheral, and none was directly central. Tan BL et al., Lamberts SWJ et al., and Chen XJ et al. were classified as peripheral. Some highly cited records addressed broad aging, frailty, oxidative-stress, nutrition, or health-outcome topics in which breast cancer and frailty appeared as indexed or contextual concepts rather than as the primary joint research question. These records were retained to preserve consistency with the predefined database-level retrieval strategy but were interpreted cautiously. Detailed definitions and record-level classifications are provided in **Supplementary Data Sheet 1**.

**Table 3:** Top 10 publications ranked by local citations among the 591 eligible WoSCC records for breast cancer and frailty research.

Rank	First author	Year	Article title	Journal	Local citations
1	Handforth C	2015	The prevalence and outcomes of frailty in older cancer patients: a systematic review	Annals of Oncology	37
2	Mandelblatt JS	2017	Frailty and long-term mortality of older breast cancer patients: CALGB 369901 (Alliance)	Breast Cancer Research and Treatment	28
3	Morgan JL	2020	Breast cancer surgery in older women: outcomes of the Bridging Age Gap in Breast Cancer Study	British Journal of Surgery	22
4	Wildiers H	2013	End points and trial design in geriatric oncology research: a joint European Organisation for Research and Treatment of Cancer–Alliance for Clinical Trials in Oncology–International Society of Geriatric Oncology position article	Journal of Clinical Oncology	21
5	Williams GR	2019	Frailty and health-related quality of life in older women with breast cancer	Supportive Care in Cancer	21
6	Ward SE	2018	Omission of surgery in older women with early breast cancer has an adverse impact on breast cancer-specific survival	British Journal of Surgery	20
7	Magnuson A	2019	Longitudinal relationship between frailty and cognition in patients 50 years and older with breast cancer	Journal of the American Geriatrics Society	19
8	Ethun CG	2017	Frailty and cancer: implications for oncology surgery, medical oncology, and radiation oncology	CA: A Cancer Journal for Clinicians	16
9	Mandelblatt JS	2018	Cancer-related cognitive outcomes among older breast cancer survivors in the Thinking and Living with Cancer Study	Journal of Clinical Oncology	16
10	Hamaker ME	2014	Baseline comprehensive geriatric assessment is associated with toxicity and survival in elderly metastatic breast cancer patients receiving single-agent chemotherapy: results from the OMEGA study of the Dutch Breast Cancer Trialists’ Group	Breast	16

Local citations were calculated within the 591-record WoSCC subset by exact DOI matching against cited-reference fields. DOI coverage was 579/591 records; 223 records received at least one matched local citation. Ties were ordered by global citation count.

**Table 2** summarizes the most globally cited records captured by the predefined search strategy within the 591-record WoSCC subset. Handforth C et al. ranked first with 838 WoSCC citations, followed by Tan BL et al. (812) and Lamberts SWJ et al. (728). Targeted manual assessment classified six of the 10 records as contextual and four as peripheral; none directly investigated the breast cancer-frailty relationship as its primary research question. Tan BL et al., Lamberts SWJ et al., and Chen XJ et al. were classified as peripheral. Citation prominence therefore did not necessarily correspond to thematic specificity. The local citation ranking in **Table 3** is more directly embedded in the retrieved breast cancer and frailty literature.

In the local citation analysis, Handforth C et al. ranked first with 37 local citations, followed by Mandelblatt JS et al. (2017, 28), Morgan JL et al. (2020, 22), Wildiers H et al. (2013, 21), Williams GR et al. (2019, 21), Ward SE et al. (2018, 20), and Magnuson A et al. (2019, 19). Three publications had 16 local citations; after tie-breaking by global citations, Ethun CG et al. ranked eighth, Mandelblatt JS et al. (2018) ninth, and Hamaker ME et al. tenth.

## 4 Discussion

### 4.1 Principal findings

This analysis mapped breast cancer and frailty research from 1973 to 2025 using an integrated final integrated dataset of 1,003 unique records from WoSCC, Scopus, and PubMed, with citation analyses restricted to 591 WoSCC records. Publication counts increased over time, although segmented regression did not identify a statistically significant change in slope after 2018. The United States, Italy, and the United Kingdom were the leading contributors after country-name harmonization. Keyword and network analyses emphasized frailty and geriatric assessment alongside treatment, toxicity, quality of life, comorbidity, and survivorship. The title/abstract audit also demonstrated that the dataset contained both directly co-mentioning publications and records retrieved through database indexing fields or broader contextual mentions, an important consideration when interpreting thematic and citation results.

### 4.2 Growth of the field and its clinical background

The higher publication counts observed in recent years coincide with growing recognition of the clinical relevance of frailty in cancer care. However, the segmented model did not support a discrete acceleration after 2018; the pattern is therefore interpreted as sustained long-term growth rather than a statistically established change-point. Breast cancer treatment is often multimodal, and patients differ substantially in physiological reserve, functional status, comorbidities, nutritional status, cognition, and treatment tolerance. Frailty provides a clinically relevant framework for characterizing vulnerability beyond conventional oncological variables. A systematic review reported substantial variation in frailty prevalence among patients with breast cancer (10). Breast cancer–specific studies have further associated frailty or geriatric deficits with chemotherapy toxicity and survival (23), surgical outcomes (24), quality of life (25), and mortality (26, 27).

The development of geriatric oncology has further strengthened interest in structured frailty and geriatric assessment. International recommendations for older adults with breast cancer emphasize integrating tumor characteristics with life expectancy, functional status, comorbidity, and patient preferences (11, 28). Multidimensional clinical factors have also been incorporated into validated models for predicting chemotherapy toxicity (29). Together, these developments illustrate why frailty assessment has become increasingly relevant to individualized treatment planning and supportive care.

The annual publication series shows sustained growth, but the formal segmented analysis did not identify a statistically significant slope change after 2018 (P = 0.465). Earlier studies often described vulnerability and frailty prevalence, whereas more recent studies increasingly address assessment tools, prediction models, interventions, and survivorship issues. This thematic shift should be distinguished from a statistically demonstrated acceleration in annual output.

However, the increase in publication volume should not be interpreted as evidence that the methodological quality or clinical applicability of the literature has improved to the same extent. Further evaluation of study designs and clinical outcomes is required.

### 4.3 Geographic distribution and collaborative structure

After harmonization of country labels, the United States ranked first in publication output, followed by Italy and the United Kingdom, with France, the Netherlands, Canada, Belgium, China, Germany, and Spain completing the top 10. The distribution confirms strong contributions from North America and Europe, while also showing an established contribution from China. Harmonizing England and other United Kingdom variants prevented artificial splitting of the United Kingdom total.

Descriptive MCP point estimates did not follow the publication-volume ranking. Germany and Belgium had the largest MCP percentages among the top 10, whereas the United States combined the largest output with the smallest point estimate in that group. Wilson 95% confidence intervals are reported in **Table 1**, and no pairwise claims of statistically significant country differences are made. The observed pattern may reflect variation in research organization, funding mechanisms, clinical-trial networks, and cross-border geriatric-oncology collaborations.

The VOSviewer country collaboration network further supports the presence of a transnational research structure. European countries formed several closely connected clusters, while North American countries, especially the United States and Canada, maintained visible links with European research groups. This pattern is clinically relevant because frailty research often requires large and diverse patient populations, as well as standardized assessment approaches. International collaboration can improve the generalizability of findings, support cross-cultural validation of frailty tools, and facilitate the development of shared guidelines for older patients with breast cancer.

However, the geographic concentration of publications also highlights an imbalance in the current evidence base. Most publications originated from Europe and North America. This is an important gap because the burden of breast cancer and population aging is increasing globally. Frailty prevalence, access to geriatric assessment, treatment resources, social support, and survivorship care may differ substantially across healthcare systems. Future studies should therefore include more diverse populations and strengthen collaboration with underrepresented regions.

### 4.4 Core contributors and publication platforms

The analysis identified Wildiers H, Cheung KL, Cohen HJ, Hurria A, Wyld L, Muss HB, Sousa P, Cunha M, Extermann M, and Horgan K as the ten most productive authors (**Figure 3A**). Their work spans geriatric assessment, systemic treatment, surgery, treatment toxicity, supportive care, and outcomes in older adults with cancer (11, 12, 23, 24, 28, 30, 31).

The leading position of the Journal of Geriatric Oncology confirms geriatric oncology as a central publication platform. Other productive journals included Cancers, Journal of Clinical Oncology, Supportive Care in Cancer, Breast Cancer Research and Treatment, Breast, Annals of Surgical Oncology, European Journal of Cancer, The Oncologist, and European Journal of Surgical Oncology. This spread across journal categories illustrates the multidisciplinary character of the field.

Institutional productivity showed a similar multidisciplinary pattern. The University of Toronto, KU Leuven, UNICANCER, Harvard University, Harvard Medical Affiliates, the University of North Carolina, Leiden University, the State University System of Florida, Leiden University Medical Center, and University Hospitals Leuven were the leading institutions in the final harmonized dataset. Their prominence reflects the importance of academic and clinical networks linking oncology, geriatrics, outcomes research, and supportive care.

The recurrent appearance of several authors and institutions suggests a degree of concentration among established research groups, although formal concentration indices were not calculated. Broader participation from additional institutions, regions, and disciplines would help expand the evidence base and promote the translation of frailty assessment into routine breast cancer care.

### 4.5 Research hotspots and conceptual structure

Keyword analysis identified “breast cancer”, “frailty”, and “geriatric assessment” as the three most frequent terms, followed by quality of life, older adults, comorbidity, frail elderly, aging, age, and chemotherapy. Because breast cancer and frailty were search concepts, their prominence was expected; the surrounding terms more directly characterize the clinical and research dimensions through which vulnerability has been studied.

The prominence of “geriatric assessment” and “comprehensive geriatric assessment” is particularly important. These terms indicate that frailty research in breast cancer is increasingly associated with structured multidimensional assessment. In older patients, frailty is rarely an isolated condition. It often coexists with multimorbidity, polypharmacy, cognitive impairment, nutritional problems, reduced mobility, and social vulnerability (12, 14, 32). Body composition and low muscle mass may provide additional information on physiological reserve and prognosis beyond body weight alone (32, 33). CGA provides a framework for identifying these vulnerabilities and incorporating them into treatment planning and supportive care strategies (12–14, 34).

The keyword co-occurrence network further revealed several major thematic clusters. One cluster was centered on treatment-related issues, including chemotherapy, adjuvant chemotherapy, endocrine therapy, surgery, radiotherapy, and treatment outcome. This suggests that a major part of the literature examines the relationships between frailty, treatment selection, treatment tolerance, and clinical outcomes. A second cluster focused on frailty, aging, older adults, geriatric assessment, and elderly patients, reflecting the importance of vulnerability assessment in older breast cancer populations. A third cluster included quality of life, survival, mortality, prognosis, comorbidity, and polypharmacy, showing that patient-centered outcomes and overall health status have become important research endpoints.

These patterns suggest that the field is moving toward a more integrated model of care. Rather than viewing breast cancer treatment as a purely tumor-directed process, current research increasingly recognizes the need to integrate oncologic efficacy with geriatric vulnerability, functional status, and patient preferences. This shift is highly relevant for clinical practice because frailty can influence the balance between the potential benefits and harms of cancer treatment, underscoring the need for structured assessment and individualized decision-making (7, 8, 11). Impaired physical functioning and frailty have also been associated with presentation with locally advanced breast cancer among older women (35). Treatment tolerance may differ substantially between fit and frail older patients (23, 31); however, frailty alone should not automatically preclude potentially beneficial treatment without structured assessment (11, 28). Prognostic models integrating tumor and patient characteristics may support more individualized primary treatment decisions in older women (36). Brief tools such as the G8 may assist in identifying older patients who could benefit from CGA (37, 38).

### 4.6 Thematic evolution and emerging frontiers

The overall CiteSpace analysis used 1995–2025 because records before 1995 were too sparse for a stable annual-slice keyword network. Its LLR labels linked comprehensive geriatric assessment and frail elderly patients with treatment-related themes such as advanced breast cancer, aromatase inhibitors, and radiation therapy, as well as physiologic and cognitive outcomes. The presence of broader oncology labels, including prostate cancer survivor and cancer patient, is consistent with the retrieved set’s database-indexing and contextual breadth.

The overall burst analysis traced a progression from adjuvant chemotherapy, functional status, and comprehensive geriatric assessment to clinical trials, breast cancer, frailty, international collaboration-related terminology, conserving surgery, older patients, and impact. The frailty burst in 2017–2018 marks a period of increased explicit attention to the concept, while the burst of “impact” extending through 2025 represents the only displayed burst active at the end of the observation period. The nonspecific term “international society” requires cautious interpretation.

The 2016–2025 analysis produced more clinically specific LLR labels, including radiation therapy, geriatric cancer patient, breast cancer survivor, HER2-negative breast cancer, geriatric assessment, aromatase inhibitor, cognitive functional outcome, and treatment-related acute toxicity. Compared with the overall map, these labels indicate increasing attention to treatment selection, survivorship, geriatric evaluation, and functional and toxicity outcomes.

The burst keyword “toxicity” (2021–2023) is noteworthy and is consistent with increased attention to treatment safety and tolerability. Breast cancer-specific prediction tools demonstrate that severe chemotherapy toxicity can be estimated using clinical and geriatric variables rather than chronological age alone (31). However, because no displayed toxicity burst extended through 2025, it is more accurate to describe toxicity as a recent period-specific focus than as an ongoing burst at the study end.

The recent LLR labels “breast cancer survivor, “ “cognitive functional outcome, “ and “treatment-related acute toxicity” point to an increased focus on outcomes beyond disease control. Physical activity and rehabilitation may help maintain function and resilience before and after treatment (39, 40), while survivorship research addresses cognitive changes (41, 42), quality of life, and supportive care needs (43). Exercise- and mind–body-based interventions have also been evaluated for fatigue, mood, inflammation, and other survivorship outcomes (44). These directions are consistent with a more preventive, personalized, and life-course-oriented framework, although the cluster labels alone do not establish intervention effectiveness.

### 4.7 Citation impact and intellectual base

Global and local citation analyses provided complementary perspectives. Several globally highly cited records addressed broader topics in oncology, aging, nutrition, oxidative stress, and frailty rather than the breast cancer-frailty interface itself. The automated title/abstract term audit identified eight of the global top 10 as frailty-only and two as mentioning both concepts; however, targeted manual assessment classified six as contextual and four as peripheral, with no record meeting the strict directly central definition. In particular, the records by Tan BL et al., Lamberts SWJ et al., and Chen XJ et al. were retrieval-eligible but thematically peripheral. Their inclusion reflects database indexing fields or broader contextual mentions, whereas their high global citation counts primarily reflect influence in broader biomedical, aging, nutrition, or health-outcome research.

Local citation analysis, by contrast, identified publications that were more directly embedded within the retrieved breast cancer and frailty literature. Highly locally cited documents were closely related to frailty assessment, geriatric assessment, treatment decision-making, surgical outcomes, treatment tolerance, cognition, quality of life, and supportive care. Some publications with modest global citation counts showed comparatively greater local influence, indicating their importance within this specific research community.

Thus, global and local citation indicators should be interpreted as complementary rather than interchangeable measures. Global citation counts identify publications with broad visibility in WoSCC, but do not establish disease-specific influence or substantive thematic centrality. Local citations more directly delineate the field-embedded intellectual base. Retrieval eligibility should therefore be distinguished from substantive thematic centrality. Accordingly, **Table 2** is presented as a transparent description of the most globally cited records captured by the predefined strategy, rather than as a definitive ranking of the most influential publications specifically investigating breast cancer and frailty.

### 4.8 Clinical and research implications

The findings of this study have several implications. First, frailty assessment has received increasing attention as a potentially important component of breast cancer care, particularly for older or otherwise vulnerable patients. The frequent appearance of terms such as “geriatric assessment”, “comprehensive geriatric assessment”, “comorbidity”, “quality of life”, and “treatment outcome” suggests that the field increasingly recognizes the need for multidimensional evaluation. In clinical practice, geriatric assessment before treatment may help clinicians identify vulnerabilities, estimate treatment risk, inform treatment intensity, and guide supportive interventions (34).

Second, the field needs more research that links frailty assessment to actionable clinical decisions. Many studies describe frailty prevalence or its association with outcomes, but future research should further clarify how frailty assessment changes treatment choices, improves patient outcomes, reduces toxicity, or enhances quality of life. Randomized evidence indicates that geriatric assessment–guided management can reduce serious treatment-related toxicity in vulnerable older adults with cancer (45, 46). These findings underscore the need to move beyond establishing the prognostic relevance of frailty toward defining how frailty information should inform clinical care. Integrating geriatric assessment into routine oncology care represents one practical approach to translating detected vulnerabilities into management recommendations (47).

Third, intervention studies are needed. The recent visibility of survivorship, cognitive functional outcomes, and treatment-related acute toxicity suggests growing interest in modifiable vulnerabilities and longer-term outcomes. Future studies should evaluate whether prehabilitation, exercise, nutritional support, medication optimization, cognitive or psychosocial interventions, social-support strategies, and multidisciplinary geriatric interventions can improve treatment tolerance and long-term outcomes in patients with breast cancer, particularly those with frailty or other vulnerabilities.

Fourth, more standardized assessment tools and outcome measures are required. Brief screening instruments such as the G8 can help identify older patients who may benefit from CGA, but screening tools should not be regarded as interchangeable with CGA (37, 38, 48). Frailty definitions and screening instruments also vary substantially across studies, which may limit comparability (5, 48). In addition, geriatric assessment may include multiple domains, such as physical function, cognition, emotional health, comorbidity, polypharmacy, nutrition, and social support (34).

Finally, the geographic imbalance in publication output suggests the need for broader international participation. Future research should include more data from underrepresented regions and healthcare systems. This is important because frailty assessment and management may be affected by resource availability, cultural factors, family support, healthcare access, and local treatment patterns.

### 4.9 Strengths and limitations

This study has several strengths. First, it integrated WoSCC, Scopus, and PubMed and retained database provenance, allowing the overlap and added contribution of each source to be quantified. Second, it applied a transparent three-stage deduplication workflow with documented normalization rules, Jaro-Winkler thresholds, manual fuzzy-match adjudication, and stage-specific audit files, yielding 1,003 unique records. Third, the complete keyword dictionary and observed merge counts are supplied. Fourth, the combined use of bibliometrix, VOSviewer, and CiteSpace enabled complementary analyses of productivity, collaboration, conceptual structure, thematic evolution, citation bursts, and citation patterns. Fifth, country labels were harmonized before all country tables and networks. Finally, four-category title/abstract audits, targeted manual assessment of the globally cited top 10, and quantitative comparison of the WoSCC subset with the full dataset make the scope and selection characteristics explicit.

Several limitations should be acknowledged. Database coverage and indexing practices differ, and only English-language articles and reviews were included. Although the search strategy required breast cancer- and frailty-related terms, some records were retrieved because relevant concepts appeared in controlled vocabulary, Keywords Plus, indexed keywords, MeSH terms, or broader contextual discussions rather than because the article directly investigated the breast cancer-frailty relationship. This issue was particularly evident among several globally highly cited general aging, oxidative-stress, nutrition, or health-outcome publications. To preserve reproducibility and avoid *post hoc* selective exclusion, records meeting the predefined database-level search, year, and document-type criteria were retained; however, a four-category title/abstract audit was performed for the full dataset and WoSCC subset, the globally cited top 10 underwent targeted manual assessment, and peripheral records were interpreted cautiously. Therefore, citation rankings and thematic maps should be understood as reflecting the literature captured by the predefined search framework rather than an exclusively clinical corpus of studies directly evaluating frailty in patients with breast cancer.

Although bibliographic fields were checked and standardized where feasible, residual variations in institution, journal, author, and keyword names may remain because records were integrated from three databases with different indexing conventions. Country labels, including England and United Kingdom variants, were harmonized for the final country analyses. Residual keyword variants, including “neoplasm” and “neoplasms”, may still have split some term frequencies.

Citation analyses were restricted to 591 WoSCC records and may not represent citation patterns in Scopus or PubMed. Although the subset closely tracked the full dataset by publication year (Spearman rho = 0.986) and country (rho = 0.911), journal-level alignment was only moderate (rho = 0.592), indicating residual selection bias. Citation counts are also influenced by publication year, journal visibility, database coverage, and field size, and bibliometric analysis identifies patterns rather than evaluating methodological quality or clinical effectiveness. Network structures, cluster assignments, and bursts depend on software settings, thresholds, and preprocessing. The VOSviewer keyword graph showed substantial thematic overlap in the separate R/Louvain diagnostic (Q = 0.2560), and LLR cluster labels are algorithm-generated descriptors rather than definitive thematic titles. Because breast cancer and frailty were search concepts, their high keyword frequencies were expected. Finally, most detected recent-period bursts ended before 2025 and should not automatically be described as currently active fronts.

## 5 Conclusion

Using a final harmonized dataset of 1,003 publications from WoSCC, Scopus, and PubMed, this study characterized the development of breast cancer and frailty research from 1973 to 2025. Publication counts increased over time, but no statistically significant slope change was detected after 2018. Thematic analyses emphasized frailty and geriatric assessment alongside treatment, tolerability, quality of life, comorbidity, functional outcomes, and survivorship. The 591-record WoSCC subset provided a database-specific citation base, and the title/abstract audit clarified the balance between direct co-mentions and broader indexed contexts. Future work should use standardized frailty terminology and assessment, expand prospective and intervention research, and strengthen international participation.

## Data Availability

The data analyzed in this study is subject to the following licenses/restrictions: The bibliographic records analyzed in this study were retrieved from the Web of Science Core Collection (WoSCC), Scopus, and PubMed. Access to WoSCC and Scopus records is subject to the respective database licensing and subscription terms; therefore, the complete source datasets cannot be publicly redistributed or included as **Supplementary Material**. Processed data supporting the findings of this study are available from the corresponding author upon reasonable request, where permitted by the applicable licenses. Requests to access these datasets should be directed to XW, wxytsf@sina.com.

## Supplementary material

Supplementary Figure 1 Top 20 countries/regions by publication output after geographic harmonization.

Supplementary Figure 2 Venn diagram of the mutually exclusive overlap among WoSCC, Scopus, and PubMed in the final 1,003-record dataset.

Supplementary Data Sheet 1 Reproducibility, validation, and audit tables, including PRISMA counts, deduplication thresholds, four-category title/abstract relevance results, targeted manual assessment of the globally cited top 10, the complete keyword dictionary and merge counts, database overlap, segmented trend analysis, country MCP confidence intervals, WoSCC subset representativeness, and network parameters.
